# Oocyte surface proteins EGG-1 and EGG-2 are required for eggshell integrity in *Caenorhabditis elegans*

**DOI:** 10.1093/g3journal/jkag013

**Published:** 2026-01-19

**Authors:** Ji Kent Kwah, Shannon Pfeiffer, Mst Gitika Khanom, Aimee Jaramillo-Lambert

**Affiliations:** Department of Biological Sciences, University of Delaware, Newark, DE 19716, United States; Department of Biological Sciences, University of Delaware, Newark, DE 19716, United States; Department of Biological Sciences, University of Delaware, Newark, DE 19716, United States; Department of Biological Sciences, University of Delaware, Newark, DE 19716, United States

**Keywords:** fertilization, egg activation, eggshell, *Caenorhabditis elegans*, EGG-1, EGG-2, WormBase

## Abstract

Metazoan eggs are surrounded by a specialized coat of extracellular matrix that mediates sperm-egg interactions. This coat is rapidly remodeled after fertilization to form a barrier that prevents polyspermy, protects against environmental insults, and provides structural support to the developing embryo. In *C. elegans*, several oocyte surface proteins have been identified that mediate these events. However, whether two of these proteins, EGG-1 and EGG-2, are required for fertilization or downstream events has been unclear. Here, we address this question using more recent advances in genome editing tools through the creation of *egg-1  egg-2* deletions of the endogenous loci. We found that *egg-1  egg-2* oocytes are fertilization competent and form rudimentary eggshells. While the integrity of the *egg-1  egg-2* eggshell is compromised and often ruptures within the uterus, some embryos can undergo several rounds of cell division. Absence of EGG-1 and EGG-2 results in the mislocalization of proteins on the embryo surface and eggshell. CBD-1, CHS-1, and MBK-2, components of the egg activation complex and outermost eggshell layer, were mislocalized, while the localization of CPG-1, a component of an inner eggshell layer, was not perturbed. Overall, our findings demonstrate that EGG-1 and EGG-2 are not required for fertilization but rather are involved in the organization of eggshell structural components and oocyte plasma membrane proteins.

## Introduction

The surface of metazoan eggs is covered by a specialized extracellular matrix (ECM) that aids in species-specific egg-sperm interactions. Following fertilization, the ECM is rapidly remodeled to prevent additional sperm entry and to protect the developing embryo (Wong and Wessel 2006). ECM remodeling occurs as part of egg activation. Egg activation is a critical process that transforms a developmentally quiescent, fertilization-competent oocyte into a fertilization-incompetent, developmentally active one-cell embryo.

In *C. elegans*, egg activation occurs immediately after fertilization and triggers a multitude of changes, including the resumption of meiosis, cortical granule exocytosis, and remodeling of the oocyte ECM. A major outcome of ECM remodeling is eggshell formation, which is essential for embryonic development. The eggshell serves multiple roles, including preventing polyspermy (when more than one sperm fertilizes a single oocyte), regulating the osmotic and chemical environment, and providing structural support to the developing embryo (Stein and Golden 2018). The *C. elegans* eggshell is composed of multiple layers, assembled in a hierarchical fashion starting with the outermost layer. The outermost three layers–the vitelline layer, chitin layer, and chondroitin proteoglycan (CPG) layer–form the trilaminar outer eggshell. These are followed by the extra-embryonic matrix, the permeability barrier, and finally the peri-embryonic space. Proper coordination between egg activation and the proteins involved in eggshell formation is essential to ensure successful embryogenesis (Olson et al. 2012; Stein and Golden 2018).

Among the proteins found on the oocyte surface, the role of EGG-1 and EGG-2 remains somewhat enigmatic. These two paralogous proteins share 67% amino acid identity and are in close proximity on chromosome III. EGG-1/2 are predicted to be LDL receptor repeat-containing proteins (Kadandale et al. 2005). EGG-1/2 localize to the oocyte plasma membrane and are endocytosed after fertilization (Kadandale et al. 2005; González et al. 2018). However, their precise roles remain unclear due to conflicting experimental results. Prior studies have reported that *egg-1/2* deficient oocytes are fertilization incompetent (Lee and Schedl 2001; Maeda et al. 2001; Kadandale et al. 2005). In contrast, Johnston et al. (2010) found that *egg-1/2* deficient oocytes are fertilization-competent but exhibit polyspermy and defects in chitin layer formation. These discrepancies may stem from technical limitations, as earlier studies relied on RNAi-mediated depletion, which can have off-target effects or inefficient mRNA knockdown. In this report, we used CRISPR/Cas9 to generate an *egg-1  egg-2* double-knockout mutant. Our findings indicate that these oocytes are fertilization competent but exhibit significant eggshell defects, including polyspermy, disrupted chitin layer formation, and impaired localization of eggshell and oocyte membrane proteins.

## Materials and methods

### *C. elegans* Strains

Standard culturing conditions were used to maintain the *C. elegans* strains used in this study (Brenner 1974). All worms were maintained at 20 °C on Modified Youngren's, Only Bacto-peptone (MYOB) plates seeded with *E. coli*  OP50. Supplementary Table 1 lists all strains used in this study and their sources. Transgenic strains expressing proteins with fluorescent tags in Fig. 5 were created by either insertion of DNA sequences at the endogenous locus (*mNeonGreen::cpg-1*, *egfp::chs-1*) or low-copy insertion at a random locus by microparticle bombardment (*cbd-1::mCherry, gfp::mbk-2*).

### CRISPR/Cas-9 mediated genome editing

CRISPR/Cas-9-mediated genome deletions were conducted using the clone-free homology-directed repair method with *dpy-10* as a co-CRISPR marker (Arribere et al. 2014; Paix et al. 2015). Design of *egg-1* and *egg-2* deletions was based on sequences from WormBase (Sternberg et al. 2024). Injections were conducted using an injection mix of 1.53 µM Cas9 protein (IDT), 6.4 µM universal tracrRNA (IDT), 1.25 µM *dpy-10* crRNA, 5 µM allele-specific crRNA, 0.92 µM *dpy-10* repair oligonucleotide, and 2.2 µM allele-specific oligonucleotide. All crRNA and oligonucleotide sequences are listed in Supplementary Table 2. Genome editing was achieved by injecting each injection mix into the gonad of young adult N2 or *+/qC1* (for the *egg-1  egg-2* double mutation) hermaphrodites. The F1 generation was screened for edits through PCR. Edited strains were verified by Sanger sequencing. Primers used for PCR and sequencing are as follows: *egg-1* forward TCGCCCAACCCTAACTTGAT, *egg-1* internal reverse TCATCCAACCTTTGCAGCAC, *egg-1* reverse CTTCGGATGTGCTGATCTGC, *egg-2* forward TACTGGTTATTTCGGCGGGA, *egg-2* internal reverse GCTGATCCATGCGATGACTG, *egg-2* reverse TTTGAACAATTCCCCTCGCG.

### Embryonic viability assay and brood sizing

Embryonic viability and brood size assays were conducted using protocols described in (Kwah and Jaramillo-Lambert 2023). Individual L4 hermaphrodites were transferred onto a single 35 mm MYOB plate spotted with *E. coli*  OP50. Each hermaphrodite was allowed to lay embryos at 20 °C for 24 h before being transferred to a new spotted 35 mm MYOB plate until cessation of embryo production. Each plate was screened for hatched larvae and unhatched embryos after 48 h. Percent embryonic viability was calculated by dividing the total hatched larvae by the total brood size (hatched larvae plus unhatched embryos).

### Widefield microscopy

The images in Figs. 2a, 3a, 3d, e, and 4a, b, and Supplementary Fig. S2 were acquired with an AxioObserver inverted widefield microscope (Carl Zeiss Inc., Gottingen, Germany) using a 20X Plan-Neofluar (numerical aperture 0.5), a 40X Plan-Neofluar (numerical aperture 1.3), or a 63X Plan-APOCHROMAT (numerical aperture 1.4) objective lens and an Axiocam 503 camera (Carl Zeiss Inc.). Each image is of a single focal plane or a projection of two focal planes (Fig. 3e). Image acquisition, processing, and analysis were conducted via Zen Microscopy software (Carl Zeiss Inc., Gottingen, Germany) and ImageJ (Fiji) (Schindelin et al. 2012). All images obtained for each respective experiment were obtained using identical parameters, with brightness and contrast adjusted for better visualization.

### Confocal microscopy

The images in Figs. 3b, c, and 5a–d were acquired with an Andor Dragonfly spinning disc confocal microscope (Oxford Instruments) using a Plan Apo 63X objective lens (numerical aperture 1.47) and a Zyla sCMOS camera (Oxford Instruments) with Z-stack intervals of 0.2 μm. Images in Fig. 2b were acquired with a Zeiss LSM980 confocal microscope using a 40× objective with Z-stack intervals of 0.21 μm. Image processing and analysis were conducted using Imaris image analysis software (Oxford Instruments). All images were obtained using identical parameters, with brightness and contrast adjusted for better visualization.

### *In utero* imaging of fertilized eggs

*egg-1(ude56) egg-2(ude52)/qC1*; *fog-2(oz40)* (control) or *egg-1(ude56) egg-2(ude52)*; *fog-2(oz40)* females were staged by picking L4 larvae and allowing them to grow into young adults overnight. *gfp::his-72; fog-2(oz40)* adult males were placed with the control or mutant female young adults and allowed to mate for one hour. After mating, the females were immobilized on a 2% agarose pad with 20 µL of 2 mM tetramisole. Imaging of live animals was conducted on an Andor Dragonfly spinning disc confocal microscope (Oxford Instruments) or on an AxioObserver inverted widefield microscope (Carl Zeiss Inc., Gottingen, Germany). For images captured with the AxioObserver widefield microscope, each sample was imaged in a single focal plane. Imaging conducted with the Andor Dragonfly spinning disc confocal microscope was a Z-stack projection with 0.5 μm steps for a total of 20 μm.

### Eggshell staining and permeability assays

L4 hermaphrodites of the indicated genotypes were plated onto fresh MYOB plates and allowed to grow for 24 h at 20 °C. Embryos were dissected from the indicated strains in egg buffer [4 mM HEPES (pH 7.4), 94 mM NaCl, 3.2 mM KCl, 2.7 mM CaCl_2_, and 2.7 mM MgCl_2_] supplemented with 16 mM of FM4-64 (Invitrogen) (Bai et al. 2020) or Calcofluor White Stain (Sigma-Aldrich) at a ratio of 1:5 Calcofluor White Stain to egg buffer. Dissection of hermaphrodites was conducted with 5 µL of supplemented egg buffer on a cover slip. A depression microscopy slide, to prevent pressure on the eggs, was fixed to the cover slip using four drops of Vaseline on the edges.

### Dissected embryo and spermatheca imaging

To image sperm in the spermatheca, L4 hermaphrodites were staged on MYOB plates and were prepared for imaging at four different time points: 8 h, 16, 24, and 48 h post-L4. 5–15 hermaphrodites were picked into 5 μL of M9 buffer on a slide, followed by whole-mount DAPI staining. To image embryos for fertilization, adult hermaphrodites 16–24 h post-L4 were dissected at the vulva to release the embryos into egg buffer, followed by whole-mount DAPI staining. For whole-mount DAPI staining, M9 or egg buffer was removed with a Kimwipe, and worms were then fixed by adding 12 μL of room temperature 100% methanol. 15 μL of 2 μg/mL DAPI was added immediately after the methanol evaporated, and the samples were covered with coverslip. Slides were incubated in the dark at room temperature for a minimum of 20 min before imaging.

### Imaging oocytes in the proximal gonad

L4 hermaphrodites were staged on MYOB plates and were prepared for imaging at three different time points: 16, 24, and 48 h post-L4. Adult hermaphrodites were immobilized on a 2% agarose pad with 20 µL of 2 mM tetramisole. For each sample, images were obtained with DIC optics in a single focal plane.

### Sperm quantification

Images used for sperm quantification were visualized in 3D using Imaris ×64 10.2.0 software, and nuclei were recognized and filtered using volume, sphericity, and voxel quantity in the surfaces function.

### Statistical analysis

Statistical analyses were performed using GraphPad Prism 6. Non-parametric Student's T-test analysis was utilized to determine the statistical significance for the embryonic viability and brood size assays. One-way ANOVA was used to determine statistical significance for sperm quantification. All experiments were conducted with a minimum of three biological replicates. Error bars indicate standard deviation.

## Results

### *egg-1* and *egg-2* null mutants are infertile

**Fig. 1.**
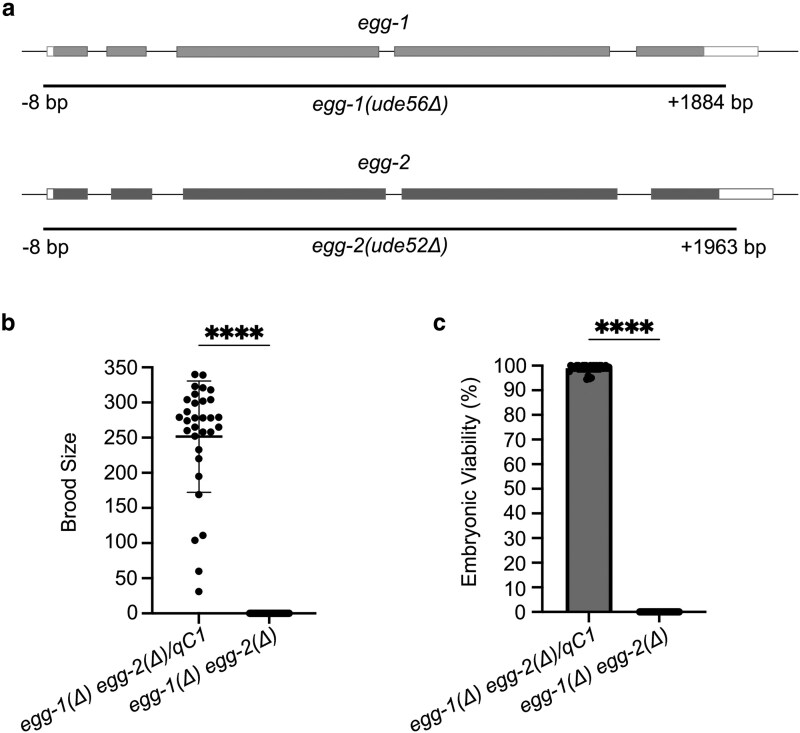
*egg-1(Δ) egg-2(Δ)* mutants do not produce viable progeny. a) Representative schematic of *egg-1* and *egg-2* gene structure. Black lines under the gene structures indicate the extent of the CRISPR/Cas9 generated deletions. b) Brood size counts for control *egg-1(ude56) egg-2(ude52)/qC1* and homozygous *egg-1(ude56) egg-2(ude52)* hermaphrodites. The brood of individual hermaphrodites was plotted from three replicate experiments. The mean brood size is indicated by the black horizontal bar. c) The percentage of viable embryos per brood was determined for both control *egg-1(ude56) egg-2(ude52)/qC1* and homozygous *egg-1(ude56) egg-2(ude52)* mutants. *P*-values for b & c were calculated by Student's T-test ****, P < 0.0001. *egg-1(ude56) egg-2(ude52)/qC1* N = 31. *egg-1(ude56) egg-2(ude52)* N = 35.

To initiate the study of the role of EGG-1 and EGG-2 in reproduction, CRISPR/Cas9 genome editing was used to delete the entire open reading frames of *egg-1* and *egg-2* individually and then both to create a double deletion strain. As prior work suggested that the double mutant is either fertilization incompetent or developmentally defective (Kadandale et al. 2005; Johnston et al. 2010), a *+/qC1* balancer strain was injected to generate the strain *egg-1(ude56) egg-2(ude52)/qC1* that can be maintained by picking heterozygous hermaphrodites (Fig. 1a). To investigate the function of EGG-1 and EGG-2 in fertilization and embryonic development, brood sizes and embryonic viability were assessed. First, we determined the brood size and embryonic viability of strains carrying single mutations of *egg-1* and *egg-2*. Wild-type (N2) worms produced an average brood size of 225.2 ± 63.0 with 98.1% viable embryos (Supplementary Fig. 1a, b). While the average brood size of *egg-2(ude52)* was not significantly different than N2 (*egg-2* = 252.0 ± 65.2), the brood size of *egg-1(ude53)* was significantly decreased (153.7 ± 43.7, Supplementary Fig. 1a). The reduced fertility phenotype was also observed in previous studies that analyzed *egg-1* RNAi and *egg-1(tm1701)*, a mutant allele with a 416 bp deletion, hermaphrodites (Kadandale et al. 2005; Johnston et al. 2010). Of the embryos produced, most were viable (*egg-1* = 98.6% and *egg-2* = 99.1% embryonic viability, Supplementary Fig. 1b). Homozygous *egg-1(ude56) egg-2(ude52)* null mutant hermaphrodites did not produce any viable progeny (Fig. 1b, c). In contrast, heterozygous *egg-1(ude56) egg-2(ude52)/qC1* hermaphrodites produced healthy populations of progeny (average brood size 251.5 ± 79.1, Fig. 1b) of viable embryos (99.0% viable, Fig. 1c). These results reconfirm that *egg-1* and *egg-2* play a role in *C. elegans* fertility.

### *egg-1 egg-2* null mutants deplete sperm more rapidly than controls

**Fig. 2.**
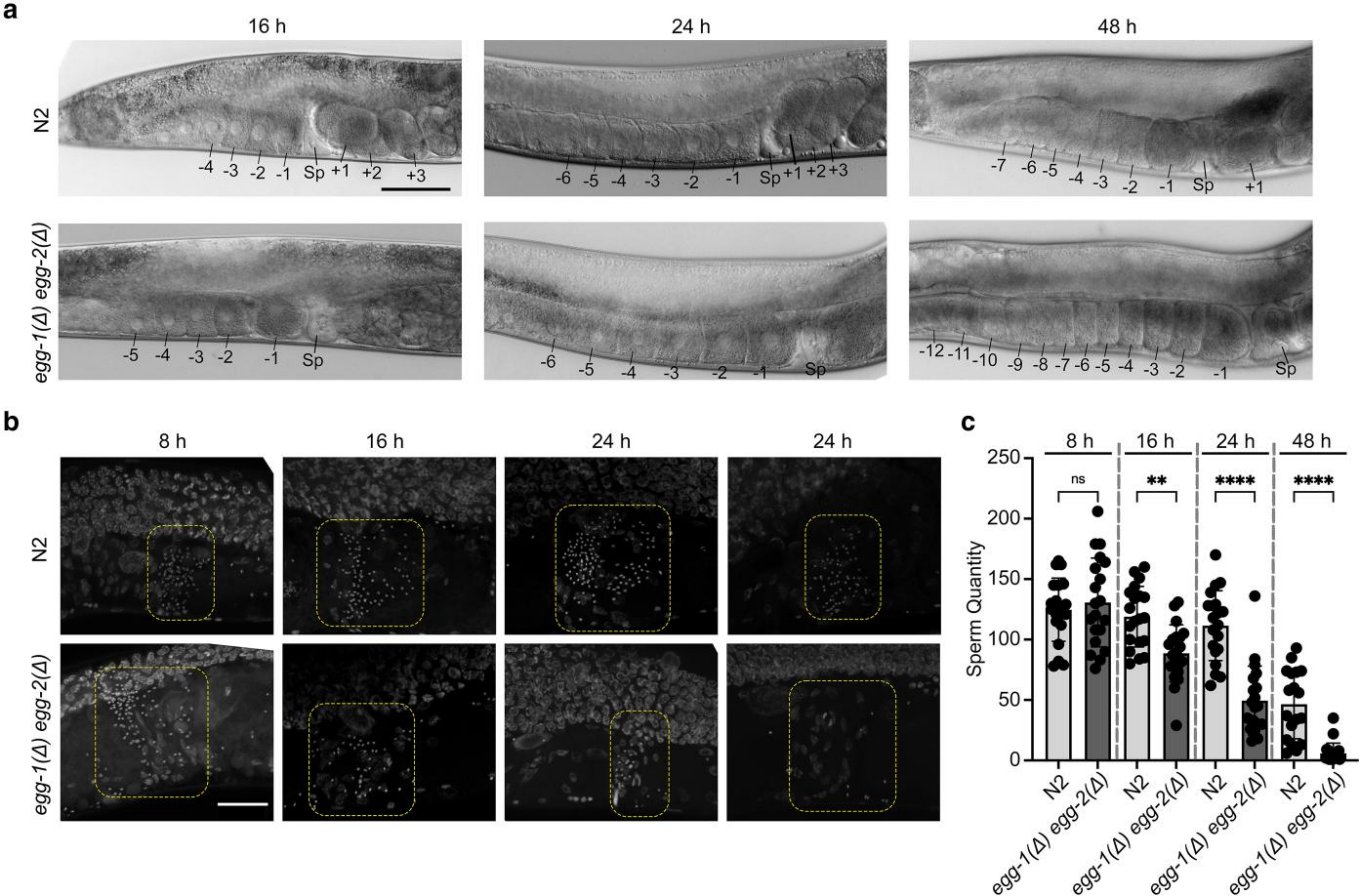
Oocyte stacking and sperm depletion in *egg-1(Δ) egg-2(Δ)* mutants. a) DIC images of the proximal gonads of wild-type (N2) and *egg-1(ude56) egg-2(ude52)* hermaphrodites. N2 N = 83; *egg-1(ude56) egg-2(ude52)* N = 61. Proximal oocytes are labeled with negative numbers (e.g., −1 to −4 in N2 at 16 h), newly fertilized embryos are indicated with positive numbers (e.g., +1 to +3 in N2 at 16 h), and Sp marks the spermatheca. By 48 h post-L4, *egg-1(ude56) egg-2(ude52)* mutant hermaphrodites have many more oocytes in the proximal gonad (>6), indicating a “stacking” phenotype. Scale bar = 50 µm. b) Images of the proximal region of DAPI stained hermaphrodite gonads. Dashed boxes indicate the region of the spermatheca where sperm are stored. Sperm is depleted more rapidly from *egg-1(ude56) egg-2(ude52)* mutants than from wild-type (N2) hermaphrodites. By 48 h post-L4, only a few spermatids are visible in the *egg-1(ude56) egg-2(ude52)* spermathecae. Scale bar = 20 µm. c) Number of sperm per individual spermathecae of adult hermaphrodites 8 h, 16, 24, and 48 h post L4. N numbers for N2 are the following: 8 h = 20, 16 h = 20, 24 h = 20, 48 h = 18. N numbers for *egg-1(ude56) egg-2(ude52)* are the following: 8 h = 20, 16 h = 20, 24 h = 20, 48 h = 20. One-way ANOVA ns = not significant, **P < 0.005, and ****P < 0.0001.

Despite the reduced fertility observed in the *egg-1(ude56) egg-2(ude52)* hermaphrodites, these worms did not have any obvious gametogenesis or somatic defects. However, we observed that oocytes in the *egg-1(ude56) egg-2(ude52)* gonad appeared to have a stacking phenotype in older adults. To investigate this further, the gonads of young adult hermaphrodites were imaged at 16, 24, and 48 h post-L4. In wild-type *C. elegans*, oocytes in the most proximal gonad are large with a cuboidal shape. The oocyte closest to the spermatheca will receive the meiotic maturation/ovulation signal from sperm in the spermatheca, where it will become rounder in shape as it matures (McCarter et al. 1999). Both wild-type (N2) and *egg-1(ude56) egg-2(ude52)* mutant gonads display normally appearing oocytes at 16 and 24 h post-L4. However, by 48 h, oocytes in *egg-1(ude56) egg-2(ude52)* mutant hermaphrodites stacked up in the gonad arm (Fig. 2a). This phenotype is associated with depletion of a sperm-provided meiotic maturation/ovulation signal (McCarter et al. 1999; Miller et al. 2001). Loss of the sperm signal can occur through several different mechanisms, including sperm motility defects or rapid consumption of sperm due to polyspermy. To determine if sperm were depleted more rapidly in *egg-1(ude56) egg-2(ude52)* mutants, we fixed and DAPI-stained whole worms and took images of spermathecae. DAPI stained sperm can be found in the spermathecae of 8 h, 16, 24, and 48 h wild-type adults (Fig. 2b). While *egg-1(ude56) egg-2(ude52)* started off with a similar number of sperm as wild-type hermaphrodites (average sperm number N2 = 124.7 vs *egg-1(ude56) egg-2(ude52)* = 130.7, *P* = 0.9269), very few sperm were observed in the spermathecae by the 24 and 48 h time points (Fig. 2c). These data demonstrate that sperm are more rapidly depleted from *egg-1(ude56) egg-2(ude52)* spermathecae potentially through an inability to return to the spermatheca after ovulation or polyspermy.

### *egg-1 egg-2* null mutants are fertilization competent

**Fig. 3.**
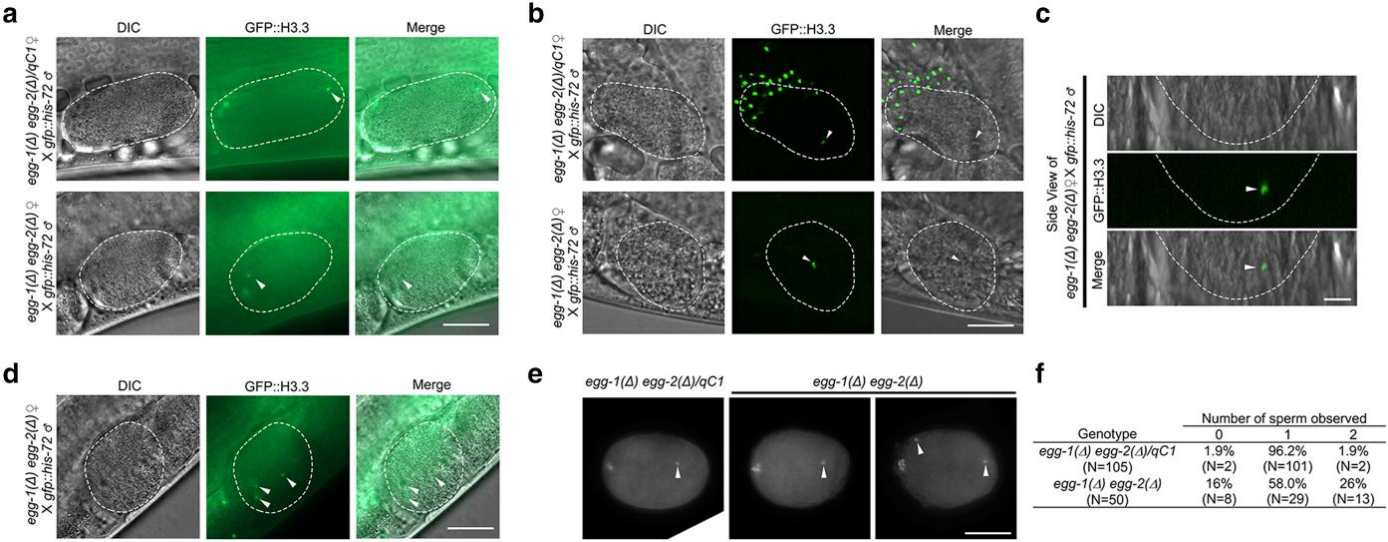
*egg-1(Δ) egg-2(Δ)* mutants are fertilization competent. a) Widefield imaging and b) spinning disc confocal microscopy of feminized control *egg-1(ude56) egg-2(ude52)/qC1; fog-2(oz40)* and homozygous *egg-1(ude56) egg-2(ude52); fog-2(oz40)* females mated with males expressing GFP::H3. Sperm chromatin observed within the newly fertilized embryo indicates successful fertilization of the *egg-1  egg-2* null oocyte. c) Side view of the fertilized *egg-1(ude56) egg-2(ude52); fog-2(oz40)* embryo in shown in 3B. d) An example of an embryo with multiple GFP::H3.3-labeled sperm chromatin foci (arrowheads). The white dashed lines outline the fertilized embryos. The total number of fertilized embryos observed per strain between the two imaging techniques: *egg-1(ude56) egg-2(ude52)/qC1; fog-2(oz40)* N = 29, and *egg-1(ude56) egg-2(ude52); fog-2(oz40)* N = 26. Scale bar = 20 µm. The scale bar for the side view panels = 10 µm. e) Embryos dissected, fixed, and DAPI stained from *egg-1(ude56) egg-2(ude52)/qC1* (N = 105 embryos) and *egg-1(ude56) egg-2(ude52)* (N = 50 embryos) hermaphrodites. Arrowheads indicate sperm chromatin. f) Table indicating the percent of embryos with 0, 1, or 2 sperm.

To evaluate the fertilization competency of *egg-1(ude56) egg-2(ude52)* null mutants, we initially attempted to mate the *egg-1(ude56) egg-2(ude52)* hermaphrodites with males expressing a GFP-tagged histone H3.3 (*gfp::his-72*) to mark paternal chromatin. However, this approach was not possible because the sperm contributed by the males were unable to migrate through the uterus of the *egg-1(ude56) egg-2(ude52)* hermaphrodites. While heterozygous hermaphrodites have a uterus filled with embryos fertilized by self-sperm, *egg-1(ude56) egg-2(ude52)* hermaphrodites have uteri filled with ooplasm as the oocytes ruptured during spermathecal transit. We believe this created an environment in which the male sperm were unable to traverse the uterus to reach the spermatheca. Therefore, we generated a feminized strain of *egg-1(ude56) egg-2(ude52)* null mutants by incorporating the *fog-2(oz40)* allele. Ovulation in *C. elegans* is triggered by a sperm-derived hormone-like signal (Miller et al. 2001). As *fog-2(oz40)* XX animals do not produce sperm, ovulations will not occur unless male sperm are provided. This ensured spermatids were able to traverse the uterus and enter the spermatheca of the *egg-1(ude56) egg-2(ude52)* mutants before the initiation of ovulation. *In utero* live imaging showed that oocytes of both *egg-1(ude56) egg-2(ude52)/qC1*; *fog-2(oz40)* heterozygous and homozygous *egg-1(ude56) egg-2(ude52)*; *fog-2(oz40)* mutants can be fertilized by male-derived sperm. GFP-labeled paternal chromatin was observed in the most recently formed one-cell embryo passing through the spermatheca, indicating successful fertilization (arrowheads, Fig. 3a). To confirm that the sperm was within the newly fertilized egg and not on the outside of the egg, three-dimensional Z-stack live *in utero* spinning disc confocal microscopy was performed. Z-stack projections confirmed the presence of the paternal GFP::H3.3-labeled sperm within newly fertilized eggs (arrowheads, Fig. 3b, c). In addition, we observed some *egg-1(ude56) egg-2(ude52)*; *fog-2(oz40)* embryos had been fertilized by multiple sperm (Fig. 3d). To assess self-fertilization and polyspermy, we dissected and DAPI-stained embryos from adult hermaphrodite uteri. After fertilization, the sperm's contributed DNA remains highly condensed near its entry point while the maternal DNA completes meiosis. We observed that 26% of *egg-1(ude56) egg-2(ude52)* embryos exhibited polyspermy (Fig. 3e, f). From these data, we conclude that EGG-1 and EGG-2 are not required for fertilization and may play a role in generating the block to polyspermy.

### EGG-1 and EGG-2 are required for eggshell integrity

**Fig. 4.**
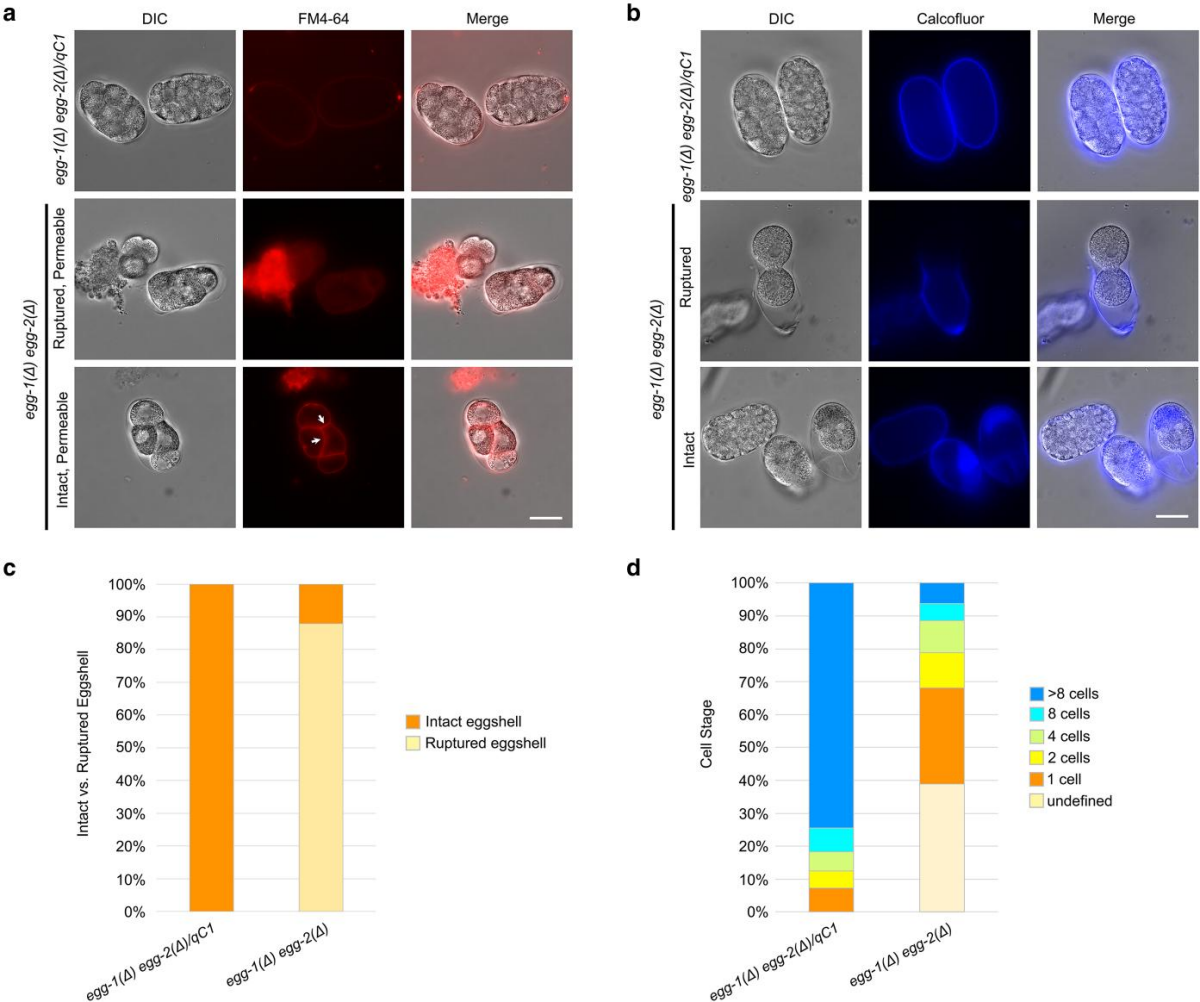
Eggshell integrity is compromised in *egg-1(Δ) egg-2(Δ)* mutants. a) FM4-64 lipophilic dye is excluded from reaching membranes in the embryo by the eggshell in control *egg-1(ude56) egg-2(ude52)/qC1* (N = 115) but permeates the *egg-1(ude56) egg-2(ude52)* mutants, staining the internal membranes of the embryo (arrows, N = 113). Scale bar = 20 µm. b) Calcofluor white stain to detect the chitin layer of the eggshell. In control *egg-1(ude56) egg-2(ude52)/qC1* a distinct chitin layer (middle row of panels) surrounds the developing embryos (N = 135). In *egg-1(ude56) egg-2(ude52)* mutant embryos, the chitin layer is present but is fragile and prone to rupture (N = 111). Scale bar = 20 µm. c) The percent of intact and ruptured embryos from *egg-1(ude56) egg-2(ude52)/qC1* and *egg-1(ude56) egg-2(ude52)* hermaphrodites. d) The percent of embryos with the indicated number of cells from *egg-1(ude56) egg-2(ude52)/qC1* and *egg-1(ude56) egg-2(ude52)* hermaphrodites. The number of embryos quantified for c-d are *egg-1(ude56) egg-2(ude52)/qC1* N = 137 and *egg-1(ude56) egg-2(ude52)* N = 113.

During the fertilization assays, we observed that embryos of *egg-1(ude56) and egg-2(ude52)* mutants were often ruptured in the uterus of the animal, indicating a defect in eggshell integrity. To further investigate if EGG-1 and EGG-2 play a role in eggshell formation similar to proteins required for egg activation, we assayed for eggshell permeability. Embryos from *egg-1(ude56) egg-2(ude52)* hermaphrodites readily incorporated the lipophilic dye FM4-64 into their plasma membranes, while this dye is excluded from *egg-1(ude56) egg-2(ude52)/qC1*, *egg-1(ude56)*, and *egg-2(ude52)* embryos which have intact eggshells (Fig. 4a, Supplementary Fig. 2a). The *egg-1(ude56) egg-2(ude52)* embryos were also osmotically sensitive with some embryos rupturing during the imaging process (Fig. 4a, middle panels). As chitin deposition is required for the major structural integrity of the embryo and other egg activation proteins are required for proper eggshell formation (Maruyama et al. 2007; Parry et al. 2009; Johnston et al. 2010 ; González et al. 2018; Tsukamoto et al. 2025), we next tested if *egg-1(ude56) egg-2(ude52)* embryos form the chitin layer of the eggshell. The eggshell of embryos from heterozygous *egg-1(ude56) egg-2(ude52)/qC1*, hermaphrodites as well as *egg-1(ude56)* and *egg-2(ude52)* single mutants stain brightly with Calcofluor white, which stains chitin (Fig. 4b, Supplementary Fig. 2b). Surprisingly, embryos from homozygous *egg-1(ude56) egg-2(ude52)* hermaphrodites also had Calcofluor white staining around the entire eggshell (Fig. 4b). This differs from previously published phenotypes of genes involved in egg activation and eggshell integrity (i.e. *cbd-1*, *spe-11*, *oops-1*), which only form a chitin cap at the site of sperm entry [see *spe-11(tn2059)*  Supplementary Fig. 2b] (Johnston et al. 2010; Tsukamoto et al. 2025).

Ruptured embryos were also observed during these assays, with some eggshells seeming to slough off the embryo (Fig. 4b middle panels). 100% of *egg-1(ude56) egg-2(ude52)/qC1* embryos had intact eggshells, while only 12% of *egg-1(ude56) egg-2(ude52)* embryos were intact (Fig. 4c). We also observed that some *egg-1(ude56) egg-2(ude52)* embryos completed several rounds of cell division without rupturing (Fig. 4a, b). Of the embryos that had some structure and did not completely disintegrate upon release from the uterus, we were unable to assign a cell stage to approximately 40% due to eggshell rupture (e.g. Embryo on the left of the middle panel of Fig. 4a). However, 61% of intact embryos were clearly definable with the majority in the one to two cell stage. While the majority of *egg-1(ude56) egg-2(ude52)/qC1* embryos made it past the eight-cell stage (74.5%), only a small percentage of *egg-1(ude56) egg-2(ude52)* had more than eight cells (6.2%) (Fig. 4d). Of note, the *egg-1(ude56) egg-2(ude52)* embryos that we observed never reached any of the morphological stages (e.g. bean, comma, 1.5-fold, etc.) and we never observed any live larvae on the plates. This phenotype differs from egg activation mutants, which fail to undergo cell division (Hill et al. 1989 ; Maruyama et al. 2007; Johnston et al. 2010; Tsukamoto et al. 2025).

### EGG-1 and EGG-2 promote even distribution of egg activation components

**Fig. 5.**
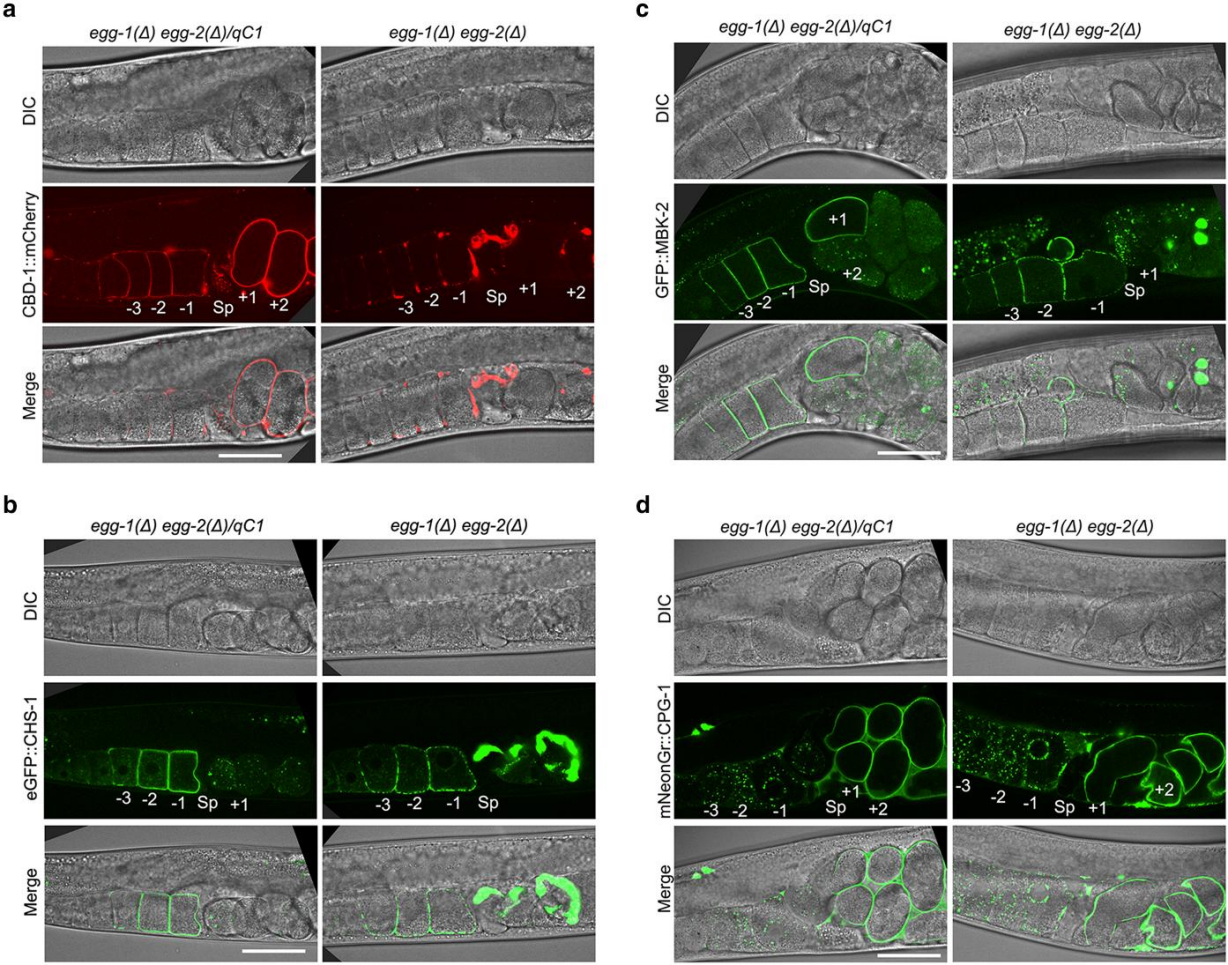
*egg-1(Δ) egg-2(Δ)* mutants exhibit defects in the cortical distribution of CBD-1, CHS-1, and MBK-2. Expression and localization of a) CBD-1::mCherry, b) eGFP::CHS-1, c) GFP::MBK-2, and d) mNeonGreen::CPG-1 in control [*egg-1(ude56) egg-2(ude52)/qC1*] and *egg-1(ude56) egg-2(ude52)*. In control, CBD-1 localizes around the oocytes and embryos, while CHS-1 and MBK-2 exhibit cortical localization in oocytes but are subsequently internalized and degraded in the embryos. In *egg-1(Δ) egg-2(Δ)*, all three of these proteins exhibit a patchy or clumping pattern in the oocytes, and localization is severely disrupted in the embryos. CPG-1 localizes as cortical granules in oocytes and then incorporates into the newly formed eggshell of embryos. A similar pattern is observed in *egg-1(Δ) egg-2(Δ)* mutants. Proximal oocytes are labeled with negative numbers, newly fertilized embryos are indicated with positive numbers, and Sp marks the spermatheca. Scale bar = 20 µm. The number of hermaphrodites examined for each strain are as follows: *egg-1(ude56) egg-2(ude52)/qC1; mcherry::cbd-1* = 26, *egg-1(ude56) egg-2(ude52); mcherry::cbd-1* = 33, *egfp::chs-1*; *egg-1(ude56) egg-2(ude52)/qC1* = 26, *egg-1(ude56) egg-2(ude52) mNGr::cpg-1* = 20, *egg-1(ude56) egg-2(ude52)/qC1; gfp::mbk-2* = 20, *egg-1(ude56) egg-2(ude52); gfp::mbk-2* = 22, *egg-1(ude56) egg-2(ude52) mNGr::cpg-1/qC1* = 24, *egg-1(ude56) egg-2(ude52) mNGr::cpg-1* = 24.

Given that loss of EGG-1 and EGG-2 resulted in eggshell defects, we sought to determine whether other oocyte surface proteins depend on EGG-1/2 for localization. Previous studies found that the chitin-binding domain protein, CBD-1, plays a pivotal role in recruiting and anchoring proteins to the oocyte membrane, including EGG-1 and EGG-2 (Johnston et al. 2010 ; González et al. 2018). Additionally, *egg-1/2* depletion via RNAi disrupts CBD-1::mCherry localization (González et al. 2018). Similar to these previous studies, we observed that *egg-1(ude56) egg-2(ude52)* mutants accumulated patches of CBD-1::mCherry on the oocyte surface and oocytes/embryos in the uterus had severely disrupted CBD-1::mCherry localization (Fig. 5a).

As previous studies found that CBD-1 is also required for the proper localization of other oocyte cortex proteins and CBD-1 localization is disrupted in *egg-1(ude56) egg-2(ude52)*, we determined the localization patterns of two additional egg cortex proteins, CHS-1 and MBK-2. CHS-1, the *C. elegans* chitin synthase, localizes in an even distribution around the oocyte plasma membrane prior to fertilization, after which it is internalized to cytoplasmic foci and degraded (Maruyama et al. 2007). In the absence of both EGG-1 and EGG-2, eGFP::CHS-1 still localizes to the oocyte plasma membrane but is found in irregular patches around the oocyte membrane and is disorganized in embryos (Fig. 5b).

MBK-2 is a kinase that regulates the oocyte-to-embryo transition by marking several maternal proteins for degradation after fertilization (Maruyama et al. 2007; Stitzel et al. 2007; Cheng et al. 2009; Parry et al. 2009). In oocytes, MBK-2 is enriched at the cortex and then localizes to subcortical foci in embryos (Maruyama et al. 2007; Stitzel et al. 2007). Similarly to CHS-1, GFP::MBK-2 localization is disrupted in *egg-1(ude56) egg-2(ude52)* oocytes with irregularly spaced patches, but is still able to form subcortical foci within embryos that remain partially intact (Fig. 5c).

Lastly, to determine if EGG-1 and EGG-2 are, in general, required for the proper localization of oocyte plasma membrane and eggshell proteins, we imaged CPG-1 tagged with mNeonGreen. CPG-1 is a protein that makes up the inner CPG layer of the eggshell (Olson et al. 2012). In oocytes, CPG-1 is found within cytoplasmic cortical granules. In embryos, cortical granule exocytosis releases CPG-1 to assemble under the chitin layer of the eggshell [Fig. 5d, (Olson et al. 2012)]. In both *egg-1(ude56) egg-2(ude52)* oocytes and embryos, mNeonGreen::CPG-1 was properly localized (Fig. 5d), indicating that EGG-1/2 is not required for the localization of inner eggshell components. Taken together, these data suggest that EGG-1 and EGG-2 organize the even distribution of a subset of oocyte plasma membrane proteins.

## Discussion

In this study, we utilized more recent genome editing techniques to create an *egg-1  egg-2* null mutant. We found that the oocyte plasma membrane proteins EGG-1 and EGG-2 are not required for fertilization competency. This differs from previous findings where knockdown of *egg-1* and *egg-2* via RNAi resulted in a sterile phenotype with unfertilized oocytes in the uterus (McCarter et al. 1999; Kadandale et al. 2005). However, in these previous experiments, RNAi targeting *egg-1* was also found to deplete *egg-2*. As there were off-target effects due to high sequence similarity, one possibility is that under these conditions, additional, yet undiscovered, *egg* genes were knocked down, causing sterility defects.

Our results support the findings of Johnston et al. 2010, which demonstrated that simultaneous RNAi knockdown of *egg-1* and *egg-2* in hermaphrodites resulted in fertilized embryos with eggshell defects (Figs. 3 and 4). However, we did observe phenotypic differences from this previous study. The Johnston et al. 2010 study found that *egg-1(RNAi) egg-2(RNAi)* animals produce fertilized embryos with severely fragmented eggshells. In this study, we found that some *egg-1(ude56) egg-2(ude52)* embryos underwent several rounds of cell division (Fig. 4). We also found that *egg-1(ude56) egg-2(ude52)* embryos formed a continuous albeit permeable eggshell (Fig. 4).

Current models place EGG-1 and EGG-2 on the surface of the oocyte plasma membrane, where it either interacts with as yet unknown sperm surface proteins or with CBD-1 (chitin binding domain protein) and the egg activation complex (EGG-3, CHS-1, EGG-4/5, and MBK-2). Our data suggest that EGG-1 and EGG-2 are not involved in fertilization; however, the results of this study cannot definitively place them in the egg activation pathway. The *egg-1(ude56) egg-2(ude52)* phenotypes we observed are less severe than *cbd-1* mutants (CBD-1 regulates eggshell assembly and anchors egg activation complex proteins) and egg activation mutants. *cbd-1* and egg activation mutants do not divide and have severely disrupted eggshells, whereas all *egg-1(ude56) egg-2(ude52)* embryos make rudimentary eggshells and some undergo cell division (Hill et al. 1989; Zhang et al. 2005; Parry et al. 2009; Johnston et al. 2010; González et al. 2018; Tsukamoto et al. 2025). We also observed examples of polyspermy (Fig. 3d–f), a feature of some egg activation mutants.

**Fig. 6.**
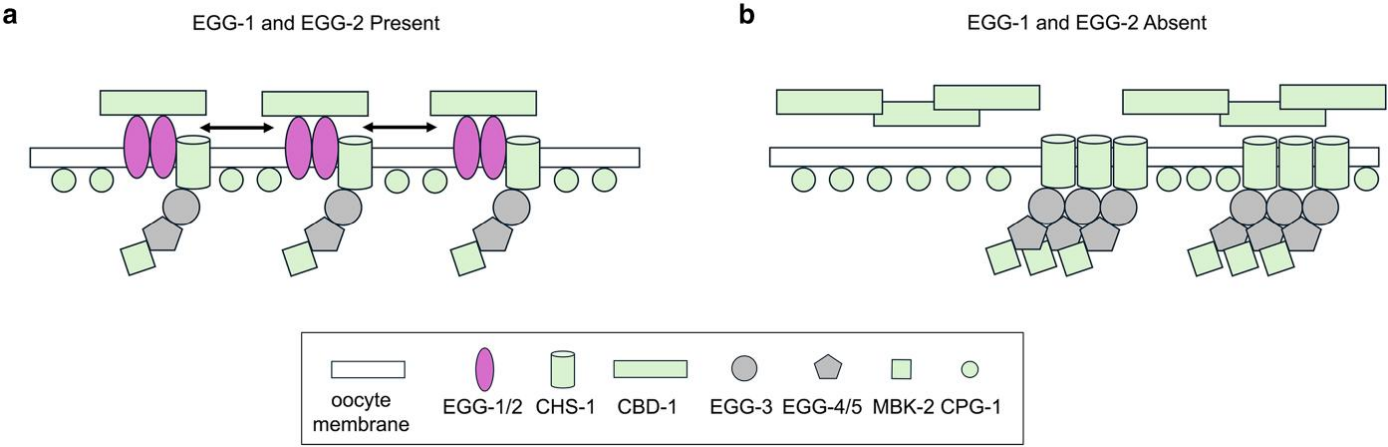
Model for the role of EGG-1/2 in organizing oocyte membrane proteins. a) EGG-1 and EGG-2 are oocyte plasma membrane proteins that function to organize and evenly distribute structural proteins (CBD-1) and the egg activation proteins (CHS-1, EGG-1-5, and MBK-2) across the oocyte surface. The regular spacing of these proteins optimizes assembly of the polyspermy block and the signaling for the transition from oocyte to embryo. Proteins represented by green-filled shapes were assessed for localization in this study. b) In the absence of EGG-1/2, CBD-1 and EGG activation complex proteins have an irregular and clumped distribution on the oocyte membrane, leading to defects in eggshell integrity and the block to polyspermy.

There is evidence that EGG-1 and EGG-2 interact with both CBD-1 and egg activation proteins. EGG-1 was not localized on the oocyte plasma membrane when *cbd-1* was knocked down (RNAi), and when *egg-1* and *egg-2* were co-depleted by RNAi, both CBD-1 and CHS-1 (chitin synthase and part of the egg activation complex) have an uneven distribution on the plasma membrane (Johnston et al. 2010; González et al. 2018). Similar to these previous studies, we found that EGG-1/2 are required for the proper localization of both embryo coat proteins (CBD-1) and components of the egg activation complex (CHS-1, MBK-2) (Fig. 5a-c). However, not all embryo coat proteins are affected, as CPG-1 localization pattern is not disrupted in the *egg-1  egg-2* null mutant (Fig. 5d). This is not surprising since CPG-1 is found in cortical granules and not on the plasma membrane prior to fertilization (Olson et al. 2012). At this time, direct protein-protein interactions have not been determined between these various components and it would be important to investigate in the future. Our data is consistent with a model where the positioning of EGG-1/2 around the oocyte plasma membrane ensures the regular distribution of CBD-1, an ECM structural protein, and the EGG activation complex (Fig. 6a) (Johnston et al. 2010; González et al. 2018). The regular spacing of these components promotes sperm interactions and assures the quick assembly of the block to polyspermy through the deposition of the chitin layer. In the absence of EGG-1/2, oocyte surface proteins do not distribute evenly across the oocyte membrane, leading to eggshell integrity defects and a failure to prevent polyspermy (Fig. 6b). Future studies to determine both the molecular activity and protein interactions of EGG-1 and EGG-2 with the various eggshell and egg activation proteins will provide crucial knowledge on how animal cells remodel egg coats after fertilization and support the transition from quiescence to active development.

## Supplementary Material

jkag013_Supplementary_Data

## Data Availability

All strains generated in this study are available upon request. The authors state that all data necessary for confirming the conclusions are presented in the article and figures. Supplementary Fig. 1 shows the brood sizes and embryonic viability of *egg-1* and *egg-2* single mutants. Supplementary Fig. 2 is FM4-64 and Calcofluor staining of *egg-1*, *egg-2*, and *spe-11* mutants. Supplementary Table 1 is a list of the *C. elegans* strains used in this study. Supplementary Table 2 contains a list of the sequences used for genome editing. Supplementary material is available online at G3: Genes, Genomes, Genetics. Supplemental material available at G3 online.
