# Supplementary material for: Oocyte surface proteins EGG-1 and EGG-2 are required for eggshell integrity in Caenorhabditis elegans
Source: G3 (Bethesda). 2026 Jan 19;16(4):jkag013. doi: 10.1093/g3journal/jkag013 (PMC13042291; doi:10.1093/g3journal/jkag013)
Supplement: jkag013_Supplementary_Data [file jkag013_supplementary_data.zip › Table_S1_G3-2025-406461.docx]

**Table S1. Strains used in this study**

| **Strain Name** | **Genotype** | **Citation/**  **Source** |
| --- | --- | --- |
| N2 | wild-type Bristol isolate | CGC |
| AJL116 | *egg-2(ude52)* III | This study |
| AJL118 | *egg-1(ude53)* III | This study |
| AJL148 | *egg-1(ude56) egg-2(ude52)/qC1* III | This study |
| AJ173 | *egg-1(ude56) egg-2(ude52)/qC1* III; *fog-2(oz40)* V | This study |
| AJL200 | *egg-1(ude56) egg-2(ude52)/qC1* III; *avIs143* [*cbd-1p::cbd-1::mCherry* + *unc-119(+)*] | This study |
| AJL202 | *egg-1(ude56) egg-2(ude52) cpg-1(tn1728[mNeonGreen::tev::3xflag::cpg-1])/qC1 III* | This study |
| AJL203 | *egg-1(ude56) egg-2(ude52)/qC1* III; *axIs1140* [*pie-1p::gfp::mbk-2* + *unc-119(+)*] | This study |
| AJL204 | *chs-1(ude35[egfp::chs-1])* I; *egg-1(ude56) egg-2(ude52)/qC1* III | This study |
| DG4915 | *his-72(uge30[gfp::his-72])* III*; fog-2(oz40)* V. | David Greenstein  UMN |
| DG5266 | *spe-11(tn2059)/tmC18* I | David Greenstein  UMN |
