# Supplementary material for: Oocyte surface proteins EGG-1 and EGG-2 are required for eggshell integrity in Caenorhabditis elegans
Source: G3 (Bethesda). 2026 Jan 19;16(4):jkag013. doi: 10.1093/g3journal/jkag013 (PMC13042291; doi:10.1093/g3journal/jkag013)
Supplement: jkag013_Supplementary_Data [file jkag013_supplementary_data.zip › Table_S2_G3-2025-406461.docx]

**Table S2. crRNAs and repair templates for mutant alleles generated in this study**

| Name | Sequence (5' to 3') |
| --- | --- |
| **Universal components** |  |
| *dpy-10* crRNA | GCUACCAUAGGCACCACGAG |
| *dpy-10* repair oligo | CACTTGAACTTCAATACGGCAAGATGAGAATGACTGGAAACCGTACCGCATGCGGTGCCTATGG TAGCGGAGCTTCACATGGCTTCAGACCAACAGCCTAT |
| **EGG-1 deletion** |  |
| *egg-1* 5’ crRNA | UCAUCAAAUUUAAAAUUUUC |
| *egg-1* 3’ crRNA | ACUUGGAGAAAUUCACAGAA |
| *egg-1* HDR repair oligo | CGCCTTTTTTCCATCTTTTCATCAAATTTAAAATTTGTGAATTTCTCCAAGTTGACCCTGAACTATTCTT |
| **EGG-2 deletion** |  |
| *egg-2* 5’ crRNA | ATTTATTCACACTATGTTTC |
| *egg-2* 3’ crRNA | AATTAGCTCTTTAGAGAGAT |
| *egg-2* HDR repair oligo | GATTTTTAAAATTTGCAAATTTATTCACACTATGTTCTCTAAAGAGCTAATTTTCATGTGAATTGTTATC |
