# Supplementary figures and images for: Oocyte surface proteins EGG-1 and EGG-2 are required for eggshell integrity in Caenorhabditis elegans
Source: G3 (Bethesda). 2026 Jan 19;16(4):jkag013. doi: 10.1093/g3journal/jkag013 (PMC13042291; doi:10.1093/g3journal/jkag013)

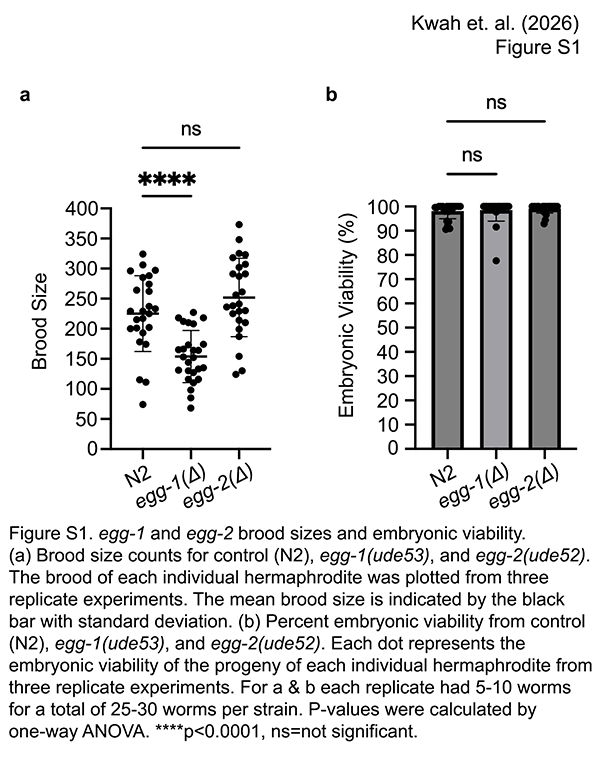

Supplement: jkag013_Supplementary_Data [file jkag013_supplementary_data.zip › Figure_S1_G3-2025-406461.tif]

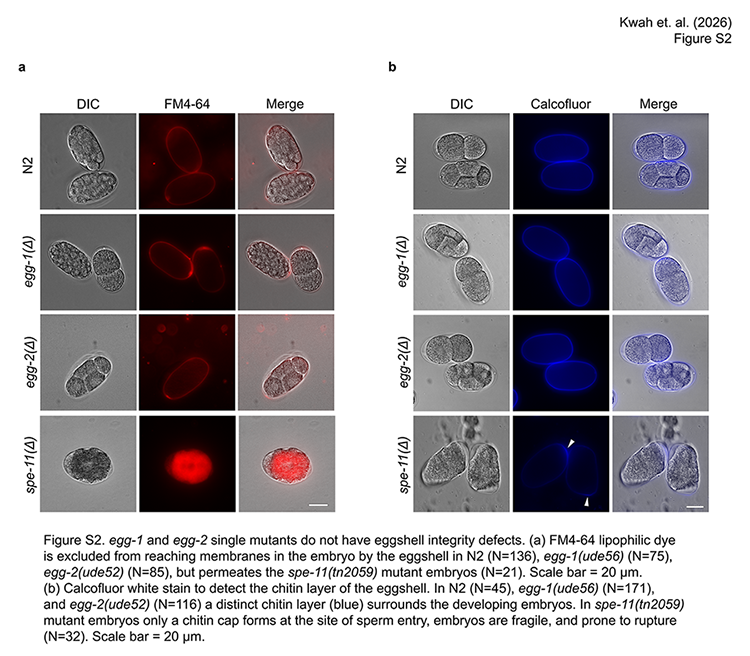

Supplement: jkag013_Supplementary_Data [file jkag013_supplementary_data.zip › Figure_S2_G3-2025-406461.tif]
